# The prognostic impact of mutations in spliceosomal genes for myelodysplastic syndrome patients without ring sideroblasts

**DOI:** 10.1186/s12885-015-1493-5

**Published:** 2015-06-27

**Authors:** Min-Gu Kang, Hye-Ran Kim, Bo-Young Seo, Jun Hyung Lee, Seok-Yong Choi, Soo-Hyun Kim, Jong-Hee Shin, Soon-Pal Suh, Jae-Sook Ahn, Myung-Geun Shin

**Affiliations:** 1 Departments of Laboratory Medicine, Chonnam National University Medical School and Chonnam National University Hwasun Hospital, 160 Ilsim-ri, Hwasun-eup, Hwasun-gun, Jeollanam-do, 519-809 South Korea; 2 College of Korean Medicine, Dongshin University, 185 Gunjaero, Naju, Jeollanam-do, 520-714 South Korea; 3 Department of Hematology-Oncology, Chonnam National University Medical School and Chonnam National University Hwasun Hospital, 160 Ilsim-ri, Hwasun-eup, Hwasun-gun, Jeollanam-do, 519-809 South Korea; 4 Brain Korea 21 Project, Center for Biomedical Human Resources, Chonnam National University Medical School, Gwangju, South Korea; 5 Environmental Health Center for Childhood Leukemia and Cancer, Chonnam National University Hwasun Hospital, 160 Ilsim-ri, Hwasun-eup, Hwasun-gun, Jeollanam-do, 519-809 South Korea

**Keywords:** SF3B1, U2AF1, SRSF2, MDS without RS

## Abstract

**Background:**

Mutations in genes that are part of the splicing machinery for myelodysplastic syndromes (MDS), including MDS without ring sideroblasts (RS), have been widely investigated. The effects of these mutations on clinical outcomes have been diverse and contrasting.

**Methods:**

We examined a cohort of 129 *de novo* MDS patients, who did not harbor RS, for mutations affecting three spliceosomal genes (*SF3B1*, *U2AF1*, and *SRSF2*).

**Results:**

The mutation rates of *SF3B1*, *U2AF1*, and *SRSF2* were 7.0 %, 7.8 %, and 10.1 %, respectively. Compared with previously reported results, these rates were relatively infrequent. The *SRSF2* mutation strongly correlated with old age (*P* < 0.001), while the mutation status of *SF3B1* did not affect overall survival (OS), progression-free survival (PFS), or acute myeloid leukemia (AML) transformation. In contrast, MDS patients with mutations in *U2AF1* or *SRSF2* exhibited inferior PFS. The *U2AF1* mutation was associated with inferior OS in low-risk MDS patients (*P* = 0.035). The *SRSF2* mutation was somewhat associated with AML transformation (*P* = 0.083).

**Conclusion:**

Our findings suggest that the frequencies of the *SF3B1*, *U2AF1*, and *SRSF2* splicing gene mutations in MDS without RS were relatively low. We also demonstrated that the *U2AF1* and *SRSF2* mutations were associated with an unfavorable prognostic impact in MDS patients without RS.

**Electronic supplementary material:**

The online version of this article (doi:10.1186/s12885-015-1493-5) contains supplementary material, which is available to authorized users.

## Background

The myelodysplastic syndromes (MDS) represent myeloid clonal hemopathies, with a relatively heterogeneous spectrum of presentation. The major clinical problems of these disorders are morbidities caused by cytopenias and the potential for MDS to evolve into acute myeloid leukemia (AML) [1]. Although cytopenias represent the major clinical challenge in low-risk disease, transformation to AML is observed in a significant number of high-risk MDS patients.

The broad range of individual genes affected by mutations indicates that a variety of molecular mechanisms are involved in the pathogenesis of MDS [2]. A number of gene mutations and cytogenetic changes have been implicated in the pathogenesis of MDS, including mutations in *RAS*, *TP53*, and *RUNX1*. However, mutations in these genes do not fully explain the pathogenesis of MDS as these mutations are also commonly found in other myeloid malignancies. In addition, approximately 20 % of MDS cases are not associated with any genetic changes. The genetic alterations responsible for dysplastic phenotypes and ineffective hematopoiesis of myelodysplasia are poorly understood [3].

A previous report by Murati *et al.* [4] described that mutations in components of the spliceosome, which are mutually exclusive, lead to splicing defects, including exon skipping, intron retention, and the use of incorrect splice sites. The consequence of mutations in spliceosomal genes is the accumulation of unspliced transcripts that affect a specific subset of mRNAs. According to Yoshida *et al.* [3] and Makishima *et al.* [5], mutations affecting spliceosomal genes that result in defective splicing could belong to a new leukemogenic pathway, and these mutations might constitute diagnostic biomarkers that could serve as therapeutic targets.

A recent study by Damm *et al.* [2] revealed that splice gene mutations are among the most frequent molecular aberrations in MDS. They might define distinct clinical phenotypes and show preferential association for mutations targeting transcriptional regulation. These genotype—phenotype associations have been demonstrated for somatic spliceosomal gene mutations in MDS with ring sideroblasts (RS). Although there have been a number of studies investigating spliceosomal mutations in MDS without RS, the effects of these mutations on clinical outcomes have not been uniform.

We investigated the prevalence and clinical impact of mutations in splicing factor 3 subunit b1 (*SF3B1*)*,* U2 small nuclear RNA auxiliary factor 1 (*U2AF1*), and serine arginine-rich splicing factor 2 (*SRSF2*) among a cohort of MDS patients without RS.

## Methods

### Patients

From 2003–2011, 129 adult patients with *de novo* MDS, diagnosed according to World Health Organization (WHO) 2008 criteria, at Chonnam National University Hwasun Hospital (Hwasun, Korea) were enrolled into this study. The patient cohort comprised 129 MDS patients without RS. A detailed summary of the enrolled patients is shown in Table 1. Of the 129 MDS patients, 58 received treatment with hypomethylating agents (42 received azacitidine and 16 received decitabine), while 11 patients underwent allogeneic hematopoietic stem cell transplantation (allo-HSCT). For the MDS patients that were treated with hypomethylating agents or allo-HSCT, this occurred prior to 2012. Therefore, we were unable to use the revised International Prognostic Scoring System (IPSS-R) [6] to decide upon treatment. Using the original International Prognostic Scoring System (IPSS), the treatment indications for hypomethylating agents or allo-HSCT were: (1) intermediate-1 with anemia, despite treatment with erythropoietin; (2) intermediate-1 with anemia accompanying other cytopenia (neutrophils < 1 × 10^3^/μl or platelets < 100 × 10^3^/μl); and (3) intermediate-2 or high risk. Azacitidine was administered subcutaneously at a dose of 75 mg/m^2^ per day for seven consecutive days, every 28 days. Decitabine was administered intravenously at a dose of 20 mg/m^2^ per day for five consecutive days, every 28 days. When we retrospectively applied the IPSS-R for treated patients (*n* = 58), 3.5, 24.1, 29.3, 29.3, and 13.8 % of patients were considered to be at very low, low, intermediate, high, and very high risk, respectively. Clinical and laboratory data for MDS patients were analyzed and reviewed, based on their electronic medical records. All enrolled MDS patients gave their written, informed consent in accordance with the Declaration of Helsinki. This study was approved by the institutional review board of Chonnam National University Hwasun Hospital.

**Table 1:** Clinical characteristics of 129 MDS patients based on the mutation status of spliceosomal genes

Characteristics	*SF3B1*^wt^ (*n* = 120, 93.0 %)	*SF3B1*^mut^ (*n* = 9, 7.0 %)	*P*	*U2AF1*^wt^ (*n* = 119, 92.2 %)	*U2AF1*^mut^ (*n* = 10, 7.8 %)	*P*	*SRSF2*^wt^ (*n* = 116, 89.9 %)	*SRSF2*^mut^ (*n* = 13, 10.1 %)	*P*
Age (years)^a^	63.4 ± 11.9	67.9 ± 19.1	0.295	63.6 ± 12.5	63.8 ± 11.8	0.975	62.8 ± 12.7	71.5 ± 5.5	0.000
Sex			0.730			0.183			0.381
Male, n (%)	67 (55.8)	4 (44.4)		63 (52.9)	8 (80.0)		62 (53.4)	9 (70.2)	
Female, n (%)	53 (44.2)	5 (55.6)		56 (47.1)	2 (20.0)		54 (46.6)	4 (30.8)	
Blood counts^a^									
WBC (× 10^3^/μl)	5.6 ± 14.3	3.7 ± 1.8	0.700	5.5 ± 14.4	5.1 ± 4.6	0.935	5.6 ± 14.6	4.2 ± 2.7	0.734
Neutrophil (× 10^3^/μl)	3.4 ± 12.0	1.5 ± 1.3	0.650	3.2 ± 12.1	3.4 ± 3.9	0.960	3.4 ± 12.3	1.9 ± 1.9	0.672
Hemoglobin (g/dl)	9.7 ± 2.2	9.2 ± 2.3	0.556	9.7 ± 2.2	8.4 ± 2.0	0.063	9.7 ± 2.3	9.4 ± 1.8	0.657
Platelet (× 10^3^/μl)	95 ± 91	168 ± 151	0.183	100 ± 98	92 ± 87	0.806	100 ± 100	91 ± 67	0.734
Bone marrow blasts (%)	5.3 ± 5.3	3.8 ± 5.0	0.398	5.0 ± 5.2	7.7 ± 6.2	0.123	5.2 ± 5.4	5.6 ± 4.3	0.783
WHO subtype, n (%)			0.303			0.516			0.094
RCUD	18 (15.0)	1 (11.1)		19 (16.0)	0 (0.0)		18 (15.5)	1 (7.7)	
RCMD	51 (42.5)	5 (55.6)		52 (43.7)	4 (40.0)		50 (43.1)	6 (46.2)	
RAEB-1	15 (12.5)	1 (11.1)		13 (10.9)	3 (30.0)		11 (9.5)	5 (38.5)	
RAEB-2	29 (24.2)	1 (11.1)		27 (22.7)	3 (30.0)		29 (25.0)	1 (7.7)	
MDS-U	1(0.8)	0 (0.0)		1 (0.8)	0 (0.0)		1 (0.9)	0 (0.0)	
MDS associated with isolated del(5q)	1 (0.8)	1 (11.1)		2 (1.7)	0 (0.0)		2 (1.7)	0 (0.0)	
Hypoplastic MDS	5 (4.2)	0 (0.0)		5 (4.2)	0 (0.0)		5 (4.3)	0 (0.0)	
Karyotype, n (%)			0.013			0.022			0.048
Normal	87 (72.5)	6 (66.7)		87 (73.1)	6 (60.0)		86 (74.1)	7 (53.8)	
-Y only	3 (2.5)	0 (0.0)		3 (2.5)	0 (0.0)		3 (2.6)	0 (0.0)	
−5 or del(5q)	2 (1.7)	1 (11.1)		3 (2.5)	0 (0.0)		3 (2.6)	0 (0.0)	
del(11q)	1 (0.8)	0 (0.0)		1 (0.9)	0 (0.0)		0 (0.0)	1 (7.7)	
del(20q)	0 (0.0)	1 (11.1)		1 (0.9)	0 (0.0)		1 (0.9)	0 (0.0)	
−7	1 (0.8)	0 (0.0)		0 (0.0)	1(10.0)		1 (0.9)	0 (0.0)	
Complex (≥3)	11 (9.2)	0 (0.0)		11 (9.2)	0 (0.0)		8 (6.9)	3 (23.1)	
Other	15(12.5)	1 (11.1)		13 (10.9)	3 (30.0)		14 (12.0)	2 (15.4)	
IPSS-R risk classification, n (%)			0.133			0.270			0.505
Very low	14 (11.8)	1 (11.1)		15 (12.6)	0 (0.0)		14 (12.1)	1 (7.7)	
Low	25 (20.8)	5 (55.6)		29 (24.4)	1 (10.0)		29 (25.0)	1 (7.7)	
Intermediate	40 (33.3)	2 (22.2)		39 (32.8)	3 (30.0)		36 (31.0)	6 (46.2)	
High	31 (25.8)	0 (0.0)		26 (21.8)	5 (50.0)		28 (24.1)	3 (23.1)	
Very high	10 (8.3)	1 (11.1)		10 (8.4)	1 (10.0)		9 (7.8)	2 (15.3)	

^a^Mean ± SD

Statistical significance is indicated by boldface type

*wt*, wild type; *mut*, mutated; *WBC*, white blood cell; *WHO*, World Health Organization; *MDS*, myelodysplastic syndrome; *RCUD*, refractory cytopenia with unilineage dysplasia; *RCMD*, refractory cytopenia with multilineage dysplasia; *MDS-U*, myelodysplastic syndrome-unclassifiable; *RAEB*, refractory anemia with excess of blasts; del, deletion; *IPSS-R*, revised International Prognostic Scoring System

### Mutation analyses of spliceosomal genes

**Fig. 1:**
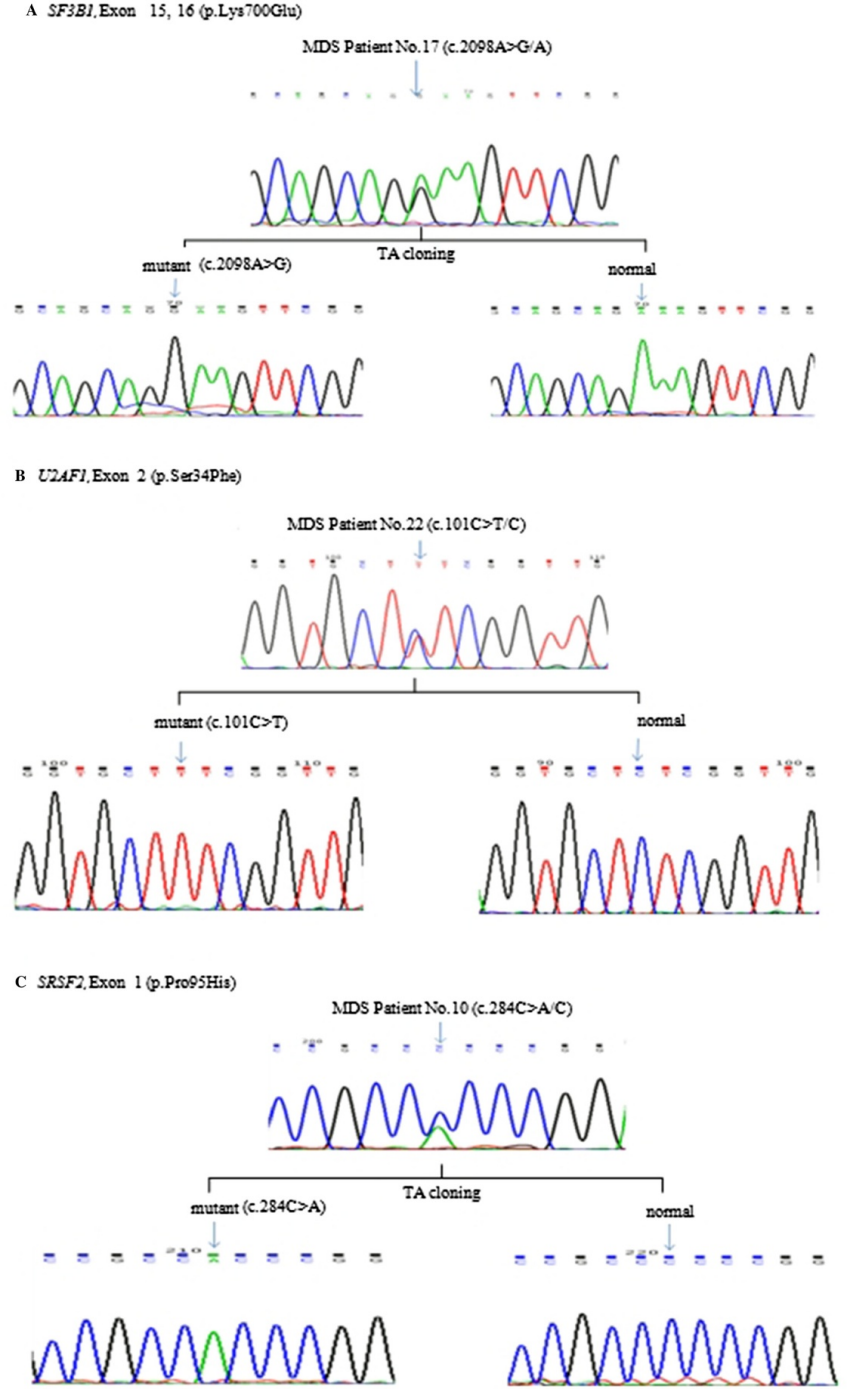
Sequencing chromatograms showing mutations in spliceosomal genes. Direct sequencing and TA cloning methods confirmed the heterozygous mutations in *SF3B1***a**, *U2AF1***b**, and *SRSF2***c**

Genomic DNA from each MDS patient was extracted using the AccuPrep Genomic DNA Extraction Kit (Bioneer, Daejeon, Korea) according to the manufacturer’s instructions. The detection of mutations in *SF3B1*, *U2AF1*, and *SRSF2* was conducted using polymerase chain reaction (PCR) followed by direct sequencing. For direct sequencing of the spliceosomal genes, six primer pairs were used (Additional file 1: Table S1) according to a published protocol (Additional file 2), with some minor modifications. Gene sequences were compared using Blast2 (http://blast.ncbi.nlm.nih.gov/Blast.cgi?PAGE_TYPE=BlastSearch&BLAST_SPEC=blast2seq&LINK_LOC=align2seq) to obtain preliminary evidence regarding polymorphisms, mutations, and for translation of amino acids. Results obtained from MDS patients were confirmed on an online database (http://genewindow.nci.nih.gov/Welcome; Additional file 2). The aberrant status of *SF3B1*, *U2AF1*, and *SRSF2*, was confirmed by TA cloning (Fig. 1) using the pGEM-T Easy vector (Promega, Madison, WI, USA). For each spliceosomal gene, three MDS patients representative of the typical heterozygous form of the gene were selected (Additional file 2).

### Cytogenetic analysis

Chromosomal analysis (G-banding) was performed on preparations from 48-h bone marrow cell cultures where mitogens were not added, according to a protocol from the American Type Culture Collection. Aberrations in chromosomes were described according to the international system for cytogenetic nomenclature 2005 and 2009.

### Statistical analyses

The *χ*^2^ test or Fisher’s exact test was performed to determine the significance of associations between *SF3B1*, *U2AF1*, and *SRSF2* mutations and other parameters, including sex, WHO classification, karyotypes, and IPSS-R risk classification. Student’s *t*-test was used to compare continuous variables such as age and hemograms. Kaplan-Meier estimation was used to plot survival curves, and log-rank tests were used to calculate the difference between survival curves. Cox proportional hazard regression analysis was used to dissect the individual impact of prognostic factors for overall survival (OS), progression-free survival (PFS), and acute myeloid leukemia (AML) transformation. All tests were two-tailed, and a *P*-value of less than 0.05 was considered statistically significant. All statistical analyses were performed using PASW version 18.0 (SPSS Inc., Chicago, IL, USA).

## Results

### Mutation status of *SF3B1*, *U2AF1*, and *SRSF2* in MDS patients

Mutations in one of the spliceosomal genes (*SF3B1*, *U2AF1*, and *SRSF2*) were observed in 24.8 % (32/129) of MDS patients . Among the 129 MDS patients, nine were identified as harboring a mutation in *SF3B1* (7.0 %), 10 patients had mutations in *U2AF1* (7.8 %), and 13 patients exhibited a mutation in *SRSF2* (10.1 %). All 129 MDS patients in this study were without RS. The *SF3B1*, *U2AF1*, and *SRSF2* mutations were mutually exclusive, with none of the patients having more than one of these genes affected (Tables 1 and 2). The mutations in *SF3B1*, *U2AF1*, and *SRSF2* were all heterozygous point mutations (*n* = 32; Table 2). The aberrant status of *SF3B1*, *U2AF1*, and *SRSF2* was confirmed by TA cloning and direct sequencing (Fig. 1).

**Table 2:** Mutations in spliceosomal genes of MDS patients and the resulting acid changes

Gene		Mutation	Amino acid change	Frequency (%)
*SF3B1*	Exon 14	c.1998G > C	p.Lys666Asn	1/129 (0.8)
		c.1986C > G	p.His662Gln	1/129 (0.8)
	Exon 15, 16	c.2098A > G	p.Lys700Glu	7/129 (5.4)
	Exon 18	No mutation	No mutation	
*U2AF1*	Exon 2	c.101C > A	p.Ser34Tyr	2/129 (1.6)
		c.101C > T	p.Ser34Phe	3/129 (2.3)
	Exon 6, 7	c.470A > C	p.Gln157Pro	5/129 (3.9)
*SRSF2*	Exon 1	c.284C > A	p.Pro95His	6/129 (4.7)
		c.284C > G	p.Pro95Arg	4/129 (3.1)
		c.284C > T	p.Pro95Leu	3/129 (2.3)

### Patient characteristics with respect to *SF3B1*, *U2AF1*, and *SRSF2* mutation status

The clinical and hematological characteristics of patients with mutated (mut) versus wild-type (wt) *SF3B1*, *U2AF1*, and *SRSF2* are shown in Table 1. Patients with *SF3B1* mutations showed significant differences in karyotype (*P* = 0.013). Positive cytogenetic findings, such as normal karyotype, −Y only, del(5q) alone, and del(20q) alone were more frequent in *SF3B1*^mut^ patients than in *SF3B1*^wt^ patients (88.9 *vs.* 75.8 %). Poor cytogenetic findings, such as complex karyotype, and abnormalities in chromosome 7 were more apparent in *SF3B1*^wt^ patients than in *SF3B1*^mut^ patients (10.0 *vs.* 0 %). There were no significant differences in age, sex, blood counts, bone marrow blasts, WHO subtype, and IPSS-R risk classification between *SF3B1*^mut^ and *SF3B1*^wt^ patients. Nevertheless, lower risk MDS patients, such as those with refractory cytopenia with unilineage dysplasia (RCUD) or refractory cytopenia with multilineage dysplasia (RCMD), were represented in higher proportions among *SF3B1*^mut^ patients than *SF3B1*^wt^ patients (66.7 *vs.* 57.5 %). For higher risk MDS patients, such as those with refractory anemia with excess blasts-1 (RAEB-1) or RAEB-2, there was a lower proportion of *SF3B1*^mut^ patients than *SF3B1*^wt^ patients (22.2 *vs.* 36.7 %).

Patients harboring mutations in *U2AF1* were mainly male (8/10) and exhibited lower hemoglobin levels (mean: 8.4 *vs.* 9.7 g/dL for *U2AF1*^mut^*vs. U2AF1*^wt^; *P* = 0.063). Our cytogenetic results revealed meaningful differences between *U2AF1*^mut^ and *U2AF1*^wt^ patients (*P* = 0.022). Positive cytogenetic findings were more frequently observed for *U2AF1*^wt^ than *U2AF1*^mut^ patients (78.3 *vs.* 60.0 %), while poor cytogenetic findings were more common in *U2AF1*^mut^ patients (10.0 *vs.* 9.2 %). In contrast, no significant differences were identified between *U2AF1*^mut^ and *U2AF1*^wt^ patients for age, sex, blood counts, bone marrow blasts, WHO subtype, and IPSS-R risk classification. The higher risk MDS patients (RAEB-1 or RAEB-2) were more likely to be *U2AF1*^mut^ patients (60.0 *vs.* 33.6 %), while lower risk MDS patients (RCUD or RCMD) were less likely to be *U2AF1*^mut^ individuals (40.0 *vs.* 59.7 %) (*P* = 0.629).

The *SRSF2*^mut^ patients were older than *SRSF2*^wt^ patients (mean: 71.5 *vs.* 62.8 years; *P* < 0.001) and mostly male (9/13). Similar to the *U2AF1*^mut^ patients, those with *SRSF2* mutations displayed a significant difference in cytogenetic results (*P* = 0.048). Good cytogenetic findings were more frequently seen for *SRSF2*^wt^ patients (79.4 *vs.* 53.8 % in *SRSF2*^mut^ patients), while poor cytogenetic findings were more common for *SRSF2*^mut^ patients (23.1 *vs.* 7.8 % in *SRSF2*^wt^ patients). We observed no significant differences in sex, blood counts, bone marrow blasts, WHO subtype, and IPSS-R risk classification between *SRSF2*^mut^ and *SRSF2*^wt^ patients. The higher risk MDS patients (RAEB-1 or RAEB-2) were more likely to be *SRSF2*^mut^ patients (46.2 *vs.* 34.5 %), while lower risk MDS patients (RCUD or RCMD) were less likely to be *SRSF2*^mut^ patients (53.9 *vs.* 58.6 %) (*P* = 0.094).

### Prognostic impact of *SF3B1*, *U2AF1*, and *SRSF2* mutations

We investigated the effects of each spliceosomal mutation on clinical outcomes. Using univariate analyses, OS and AML transformation rates according to the mutation status of the three genes were not significant (Table 3). An inferior PFS was seen for *U2AF1*^mut^ patients (HR = 4.409; 95 % CI, 1.174–16.558; *P* = 0.033) and *SRSF2*^mut^ patients (HR = 3.878; 95 % CI, 1.181–12.726; *P* = 0.018).

**Table 3:** Univariate analysis for overall survival (OS), progression-free survival (PFS), and AML transformation^a^

	OS			PFS			AML transformation		
	HR	95 % CI	*P*	HR	95 % CI	*P*	HR	95 % CI	*P*
Age (>60 years *vs.* ≤ 60 years)	0.964	0.374–2.487	0.940	1.295	0.516–3.252	0.581	0.924	0.290–2.945	0.893
IPSS-R risk groups^b^, higher *vs.* lower	5.600	1.453–21.583	0.010	5.864	1.186–28.982	0.023			
*SF3B1*^c^ (mut *vs.* WT)	1.347	0.261–6.947	0.662	0.452	0.054–3.779	0.684			
*U2AF1* (mut *vs.* WT)	1.167	0.231–5.893	1.000	4.409	1.174–16.558	0.033	0.906	0.106–7.737	1.000
*SRSF2* (mut *vs.* WT)	0.823	0.170–3.989	1.000	3.878	1.181–12.726	0.018	2.864	0.684–11.989	0.151

Statistical significance is indicated by boldface type

^a^Univariate analysis of OS, PFS, and AML transformation was performed by two-sided Fisher’s exact test or *χ*^2^ test

^b^IPSS-R higher indicates very high risk or high risk, and IPSS-R lower indicates low risk or very low risk

^c^For the IPSS-R lower risk group or *SF3B1*^mut^ patients, no AML transformation was found

AML, acute myeloid leukemia; CI, confidence interval; HR, hazard ratio; IPSS-R, revised International Prognostic Scoring System; mut, mutated; WT, wild-type

The IPSS-R was used to derive clinical prognosis for MDS. To establish whether the mutation status of spliceosomal genes can add to the predictive power of IPSS-R, we performed multivariable Cox regression analyses, examining age, sex, IPSS-R total score, and *SF3B1*/*U2AF1*/*SRSF2* mutation status (Table 4). The IPSS-R total score strongly correlated with OS, PFS, and AML transformation, while the mutation status of *U2AF1* (HR = 4.840; 95 % CI, 1.655–14.157; *P* = 0.004) and *SRSF2* (HR = 4.379; 95 % CI, 1.604–11.952; *P* = 0.004) remained an independent predictor for PFS. AML transformation was not associated with the mutation status of *SF3B1*.

**Table 4:** Cox regression analysis for overall survival (OS), progression-free survival (PFS), and AML transformation^a^

	OS			PFS			AML transformation		
	HR	95 % CI	*P*	HR	95 % CI	*P*	HR	95 % CI	*P*
Age (years)	1.029	0.987–1.073	0.174	1.039	0.996–1.084	0.074	1.007	0.952–1.064	0.819
Sex (male *vs.* female)	0.711	0.298–1.695	0.442	0.881	0.388–1.999	0.761	0.823	0.266–2.553	0.737
IPSS-R total score	1.634	1.263–2.115	<0.0001	1.546	1.214–1.969	<0.0001	1.699	1.200–2.405	0.003
*SF3B1*^b^ (mut *vs.* WT)	2.663	0.572–12.397	0.212	1.533	0.193–12.145	0.686			
*U2AF1* (mut *vs.* WT)	1.648	0.365–7.436	0.516	4.840	1.655–14.157	0.004	1.252	0.149–10.494	0.836
*SRSF2* (mut *vs.* WT)	1.216	0.270–5.485	0.799	4.379	1.604–11.952	0.004	2.672	0.697–10.245	0.152

Statistical significance is indicated in boldface type

^a^Multivariate analysis of OS, PFS, and AML transformation was performed using a Cox proportional hazards regression model that included age, sex, IPSS-R total score, and mutation status of *SF3B1*, *U2AF1*, and *SRSF2*

^b^For *SF3B1*^mut^ patients, no AML transformation was seen

AML, acute myeloid leukemia; CI, confidence interval; HR, hazard ratio; IPSS-R, revised International Prognostic Scoring System; mut, mutated; WT, wild-type

**Fig. 2:**
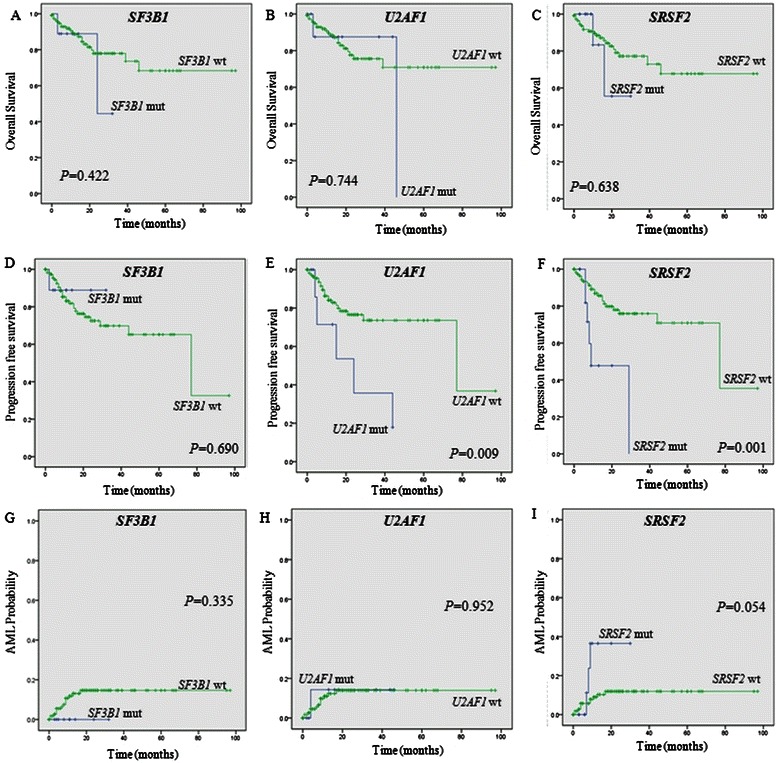
Clinical outcomes are affected by the mutation status of spliceosomal genes. Kaplan-Meier analysis of overall survival **a**–**c**, progression-free survival **d**–**f**, and probability of AML transformation **g**–**i** for the total MDS patient cohort (*n* = 129), stratified according to *SF3B1*, *U2AF1*, and *SRSF2* mutation status. AML, acute myeloid leukemia; wt, wild-type; mut, mutant

**Fig. 3:**
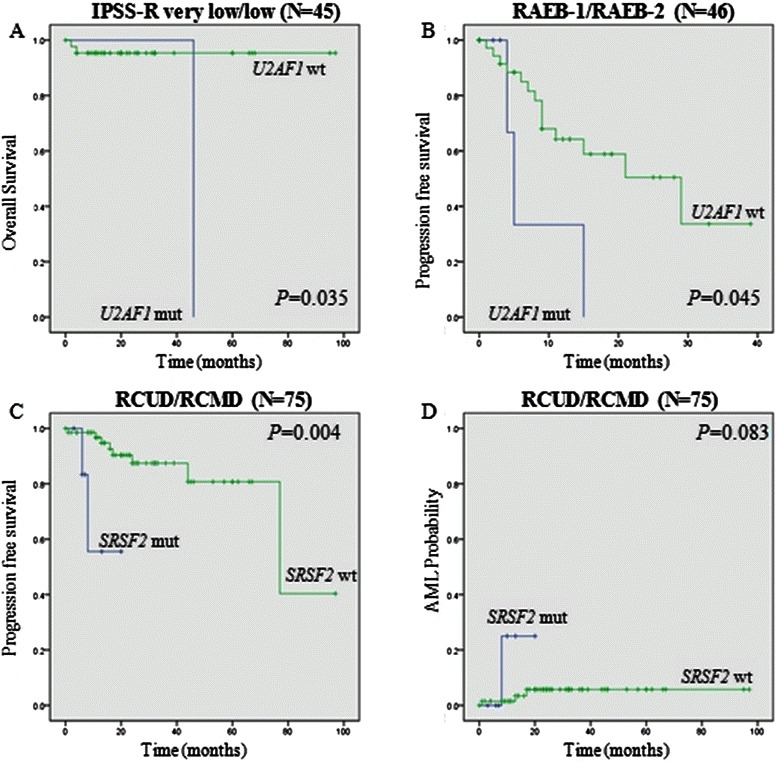
Impact of *U2AF1***a**–**b** and *SRSF2***c**–**d** mutations on the clinical outcome of the MDS subgroups. Overall survival **a** and progression-free survival **b**–**c** are affected by *U2AF1* or *SRSF2* genotypes according to subgroup analysis of MDS patients. The probability of AML progression was increased for RCUD and RCMD patients with a mutation in *SRSF2***d**. AML, acute myeloid leukemia; IPSS-R, revised International Prognostic Scoring System; mut, mutant; RAEB, refractory anemia with excess blasts; RCMD, refractory cytopenia with multilineage dysplasia; RCUD, refractory cytopenia with unilineage dysplasia; wt, wild-type

We evaluated OS, PFS, and AML probabilities according to the mutation status of spliceosomal genes in all MDS patients (Fig. 2a–i), and subgroups of MDS patients (Fig. 3a–d), using Kaplan-Meier estimation. No differences in survival were seen for all MDS patients with or without mutations in *SF3B1* (Fig. 2a, d, and g). Patients carrying a mutation in *U2AF1* (*P* = 0.009; Fig. 2e) or *SRSF2* (*P* = 0.001; Fig. 2f) exhibited significantly lower PFS compared with wild-types. The presence of a *SRSF2* mutation was a somewhat unfavorable prognostic factor for AML transformation (*P* = 0.054; Fig. 2i).

MDS subgroup analysis revealed that the poor impact of a *U2AF1* mutation on OS was only demonstrated in the lower risk groups (very low and low) defined by IPSS-R (*P* = 0.035; Fig. 3a). In addition, patients harboring the *U2AF1* mutation showed inferior PFS in the higher risk groups (RAEB-1 or RAEB-2) defined by WHO 2008 criteria (*P* = 0.045; Fig. 3b). Patients with the *SRSF2* mutation showed inferior PFS in the lower risk groups (RCUD or RCMD) defined by WHO 2008 criteria (*P* = 0.004; Fig. 3c). Patients with a *SRSF2* mutation exhibited a somewhat increased rate for AML transformation among lower risk (RCUD or RCMD) MDS patients (*P* = 0.083; Fig. 3d). No survival differences were seen between MDS patients with or without the *SF3B1* mutation (data not shown).

## Discussion

Recent reports regarding whole exome sequencing in MDS patients by Yoshida *et al.* [3] and Papaemmanuil *et al.* [7] suggest that spliceosome mutations have some clinical relevance. Identifying the impact of these mutations on MDS pathogenesis holds some promise for the therapeutic modulation of mRNA splicing [8]. The exact functional consequences of these spliceosomal mutations in MDS pathogenesis and other hematological malignancies remain largely unknown, and are being intensely investigated [9]. The molecular diversity of MDS corresponds to the clinical and phenotypic heterogeneities of these syndromes. Moreover, molecular defects could potentially serve as biomarkers for the identification of therapeutic targets [5]. To date, these genotype–phenotype associations of MDS have been described in many previous studies. Numerous researchers have investigated spliceosomal mutations in MDS without RS; however, the effects of these mutations on clinical outcomes have not been uniform. We investigated the prevalence and prognostic implication of the *SF3B1, U2AF1*, and *SRSF2* mutations in MDS patients without RS from Korea.

Our findings indicate that the *SF3B1, U2AF1*, and *SRSF2* mutations were relatively infrequent in MDS patients without RS, contradicting the results from a previous study. In addition, our results demonstrate that the *U2AF1* and *SRSF2* mutations, unlike *SF3B1*, were associated with a negative prognostic impact for MDS patients without RS.

Spliceosomes in the nucleus are complexes composed of small nuclear RNAs (snRNA) and numerous protein subunits. These spliceosomes serve to remove introns from genes that encode proteins [10]. Identifying these genes and understanding the mechanisms involved in aberrant splicing might lead to advancements in diagnosis and treatment of MDS and other diseases [11]. According to a recent report by Makishima *et al.*, mutations affecting spliceosomal genes that result in defective splicing belong to a new leukemogenic pathway, with these mutations possibly constituting diagnostic biomarkers that could be therapeutic targets [5].

These spliceosomal gene mutations occur at varying frequencies for different disease subtypes, and contribute to differences in survival outcomes [9]. The *SF3B1* gene is located on chromosome 2q33.1 and codes for the *SF3B1* protein complex, which is involved in the early stages of spliceosome assembly. *U2AF1* gene is located on chromosome 21q22, and encodes proteins that play a role in the early steps of 3′ splice site recognition. The *SRSF2* gene is located on chromosome 17q25.2, with the coding protein known to play a role in preventing exon skipping and ensuring the accuracy of splicing [12].

It was previously reported that the incidence of MDS with RS is far less common than that of MDS without RS in the Korean population [13, 14]. Consistent with previous studies, our study population comprised 129 MDS patients without RS. For this cohort, the mutation rates of *SF3B1*, *U2AF1*, and *SRSF2* were 7.0, 7.8, and 10.1 %, respectively (Table 1). The occurrence of mutations in these genes, for MDS patients without RS, were relatively infrequent compared with that seen in earlier studies [3, 8, 9, 14]. Hahn and Scott reported that the p.Lys700Glu was the most recurrently occurring alteration in both MDS and chronic lymphocytic leukemia [10]. In the current study, this particular mutation was the most common seen in spliceosomal genes likewise (Table 2).

Malcovati *et al.* reported that only 5.3 % (2/38) of patients with AML evolving from MDS carried a somatic mutation in *SF3B1* [15]. In our current study, none of the *SF3B1*^mut^ MDS patients progressed into AML, and these patients were more likely to present with advantageous cytogenetic findings. However, *U2AF1*^mut^ and *SRSF2*^mut^ patients were considered to belong to higher risk MDS groups or to have a poor cytogenetic findings (Table 1).

We also found that the *U2AF1* mutation mainly occurred in males and correlated with relatively low hemoglobin levels. It was previously that mutations in *U2AF1* confer the suppression of growth *in vitro* [3], possibly contributing to the cytopenias seen in *U2AF1*^mut^ patients within the current MDS cohort. Occurrence of the *SRSF2* mutation strongly correlated with older individuals (*P* < 0.001), similar to the findings of Wu *et al.* [16] (Table 1).

We found that the IPSS-R total score had a strong association with OS, PFS, and AML transformation (Table 4). However, the prognostic impact of spliceosome gene mutations in MDS patients remains controversial [16]. Some studies have reported that *SF3B1* mutations are a marker of favorable outcomes for MDS [7, 15]. However, results from other studies [17], including our analysis in the current study, indicate that *SF3B1* mutations do not represent an independent prognostic factor (Tables 3 and 4, Fig. 1). These differences could be attributed to the heterogeneity of the disease itself, the composition of patient populations, and the various treatment strategies used [17, 18].

Regarding the *U2AF1* mutation, results from one study concluded that it did not influence OS [19], while another report claimed that it was associated with shorter OS [5]. Analysis of our whole cohort, or even subgroup analysis of MDS patients, revealed inferior OS and PFS for *U2AF1*^mut^ patients (Figs. 2e and 3a–b). This negative prognostic impact for PFS was also seen when we conducted univariate or multivariate Cox regression analysis (Tables 3 and 4), further supporting the idea that the *U2AF1* mutation could be an independent prognostic marker for MDS.

The *SRSF2* mutation negatively affected PFS in MDS patients, especially for those in the lower risk MDS groups (Figs. 2f and 3c). We also found that the *SRSF2* mutation was an independent prognostic factor for a poor PFS outcome (Tables 3 and 4). Consistent with findings by Thol *et al*., who reported that *SRSF2* mutations were associated with an increased risk of progression to AML [18], we observed a somewhat significant impact of the *SRSF2* mutation on the progression time to AML transformation (Figs. 2i and 3d). In a previous study, deletion of *SRSF2* contributes to genomic instability, which is a predictive marker for adverse outcomes in MDS, and possibly explains why *SRSF2* mutations confer a strong adverse effect [18].

## Conclusions

In summary, we observed that mutations in *SF3B1, U2AF1*, and *SRSF2*, in MDS patients without RS, were relatively infrequent molecular events. The mutation status of *SF3B1* was not associated with OS, PFS, or AML transformation, regardless of the groupings used in our analyses. In contrast, all *U2AF1*^mut^ and *SRSF2*^mut^ patients displayed inferior PFS. We observed that mutations in *U2AF1* were associated with inferior OS in the lower risk MDS groups defined by IPSS-R (very low or low risk) and that there was somewhat of an association between AML transformation and mutations in *SRSF2*.

## Additional files

Additional file 1: **Table S1.** Primers for PCR and direct sequencing of spliceosomal genes.
Additional file 2:
**Supplementary methods for sequencing of each spliceosomal gene and the confirmation of aberrant status.**

## Abbreviations

MDS: Myelodysplastic syndrome
RS: Ring sideroblasts
OS: Overall survival
PFS: Progression free survival
IPSS-R: Revised-International Prognostic Scoring System
SF3B1: Splicing factor 3 subunit b1
U2AF1: U2 small nuclear RNA auxiliary factor 1
SRSF2: Serine arginine-rich splicing factor 2
WHO: World Health Organization
PCR: Polymerase chain reaction
AML: Acute myeloid leukemia
mut: Mutated type
wt: Wild type
RCUD: Refractory cytopenia with unilineage dysplasia
RCMD: Refractory cytopenia with multilineage dysplasia
RAEB: Refractory anemia with excess blasts

## References

[1] Greenberg PL, Attar E, Bennett JM, Bloomfield CD, De Castro CM, Deeg HJ (2011). Myelodysplastic syndromes. J Natl Compr Canc Netw.

[2] Damm F, Kosmider O, Gelsi-Boyer V, Renneville A, Carbuccia N, Hidalgo-Curtis C (2012). Mutations affecting mRNA splicing define distinct clinical phenotypes and correlate with patient outcome in myelodysplastic syndromes. Blood.

[3] Yoshida K, Sanada M, Shiraishi Y, Nowak D, Nagata Y, Yamamoto R (2011). Frequent pathway mutations of splicing machinery in myelodysplasia. Nature.

[4] Murati A, Brecqueville M, Devillier R, Mozziconacci MJ, Gelsi-Boyer V, Birnbaum D (2012). Myeloid malignancies: mutations, models and management. BMC Cancer.

[5] Makishima H, Visconte V, Sakaguchi H, Jankowska AM, Kar SA, Jerez A (2012). Mutations in the spliceosome machinery, a novel and ubiquitous pathway in leukemogenesis. Blood.

[6] Greenberg PL, Tuechler H, Schanz J, Sanz G, Garcia-Manero G, Solé F (2012). Revised international prognostic scoring system for myelodysplastic syndromes. Blood.

[7] Papaemmanuil E, Cazzola M, Boultwood J, Malcovati L, Vyas P, Bowen D (2011). Somatic SF3B1 mutation in myelodysplasia with ring sideroblasts. New England J Med.

[8] Abdel-Wahab O, Levine R (2011). The spliceosome as an indicted conspirator in myeloid malignancies. Cancer Cell.

[9] Visconte V, Makishima H, Maciejewski JP, Tiu RV (2012). Emerging roles of the spliceosomal machinery in myelodysplastic syndromes and other hematological disorders. Leukemia.

[10] Hahn CN, Scott HS (2012). Spliceosome mutations in hematopoietic malignancies. Nature genetics.

[11] Padgett RA (2012). New connections between splicing and human disease. Trends Genet.

[12] Maciejewski JP, Padgett RA (2012). Defects in spliceosomal machinery: a new pathway of leukaemogenesis. Br J Haematol.

[13] Jung S-W, Lee S-Y, Jekarl D-W, Kim M, Lim J, Kim Y (2011). Cytogenetic characteristics and prognosis analysis in 231 myelodysplastic syndrome patients from a single institution. Leuk Res.

[14] Je EM, Yoo NJ, Kim YJ, Kim MS, Lee SH (2013). Mutational analysis of splicing machinery genes SF3B1, U2AF1 and SRSF2 in myelodysplasia and other common tumors. Int J Cancer J Int Du cancer.

[15] Malcovati L, Papaemmanuil E, Bowen DT, Boultwood J, Della Porta MG, Pascutto C (2011). Clinical significance of SF3B1 mutations in myelodysplastic syndromes and myelodysplastic/myeloproliferative neoplasms. Blood.

[16] Wu SJ, Kuo YY, Hou HA, Li LY, Tseng MH, Huang CF (2012). The clinical implication of SRSF2 mutation in patients with myelodysplastic syndrome and its stability during disease evolution. Blood.

[17] Damm F, Thol F, Kosmider O, Kade S, Löffeld P, Dreyfus F (2012). SF3B1 mutations in myelodysplastic syndromes: clinical associations and prognostic implications. Leukemia.

[18] Thol F, Kade S, Schlarmann C, Löffeld P, Morgan M, Krauter J (2012). Frequency and prognostic impact of mutations in SRSF2, U2AF1, and ZRSR2 in patients with myelodysplastic syndromes. Blood.

[19] Graubert TA, Shen D, Ding L, Okeyo-Owuor T, Lunn CL, Shao J (2012). Recurrent mutations in the U2AF1 splicing factor in myelodysplastic syndromes. Nat Genet.

