# Supplementary material for: The prognostic impact of mutations in spliceosomal genes for myelodysplastic syndrome patients without ring sideroblasts
Source: BMC Cancer. 2015 Jun 27;15:484. doi: 10.1186/s12885-015-1493-5 (PMC4483202; doi:10.1186/s12885-015-1493-5)
Supplement: Additional file 1: — Table S1. Primers for PCR and direct sequencing of spliceosomal genes. [file 12885_2015_1493_MOESM1_ESM.docx]

| **Supplementary Table 1. Primers for PCR amplification and direct sequencing of spliceosomal genes** | | | | |
| --- | --- | --- | --- | --- |
| Gene | | Direction | Sequence (5′→ 3′) | Size^*^ |
| *SF3B1* | Exon 14 | Forward | TAGAGTGGAAGGCCGAGAGA | 1.2 kb |
|  |  | Reverse | TTCAAGAAAGCAGCCAAACC |  |
|  | Exon 15, 16 | Forward | GTTGATATATTGAGAGAATC | 536 bp |
|  |  | Reverse | TTTAAAATTCTGTTAGAACC |  |
|  | Exon 18 | Forward | CGATGTTTGGTCACTTTTCT | 462 bp |
|  |  | Reverse | TTGCTTGACAACTAATATGC |  |
| *U2AF1* | Exon 2 | Forward | TGCTGCTGACATATTCCATGT | 400 bp |
|  |  | Reverse | AGTCGATCACCTGCCTCACT |  |
|  | Exon 6, 7 | Forward | ATTAAAGCGTGGATGGCAAG | 524 bp |
|  |  | Reverse | TCCAAAGAGGACATTTGGAT |  |
| *SRSF2* | Exon 1 | Forward | GTGGACAACCTGACCTACCG | 327 bp |
|  |  | Reverse | CCTCAGCCCCGTTTACCT |  |
| ^*^Size of the amplified product for the *SF3B1*, *U2AF1*, and *SRSF2* gene. | | | | |
