# Supplementary material for: The prognostic impact of mutations in spliceosomal genes for myelodysplastic syndrome patients without ring sideroblasts
Source: BMC Cancer. 2015 Jun 27;15:484. doi: 10.1186/s12885-015-1493-5 (PMC4483202; doi:10.1186/s12885-015-1493-5)
Supplement: Additional file 2: — Supplementary methods for sequencing of each spliceosomal gene and the confirmation of aberrant status. [file 12885_2015_1493_MOESM2_ESM.docx]

**Supplementary Methods**

**1. Sequencing of the splicing genes**

Patient DNA was amplified using the following PCR conditions: 96 ºC for 1 min and 35 cycles of 96 ºC for 30 s, 58.8 ºC for 50 s, and 72 ºC for 1 min, followed by 1 cycle of 72 ºC for 5 min. Purified PCR fragments were sequenced directly using forward or reverse primers (Supplementary Table 1). Each amplified splicing gene product was purified using the AccuPrep PCR Purification Kit (Bioneer) and sequenced with a BigDye Terminator v3.1 Ready Reaction Kit (Applied Biosystems, Foster City, CA, U.S.A.).

**2. Splicing gene sequences confirmation**

The sequences were compared with the reference sequences available from NCBI FASTA in which transcript *SF3B1* NCBI Reference Sequence: NC_000002.11; transcript *U2AF1* NCBI Reference Sequence: NC_000021.8; transcript *SRSF2* NCBI Reference Sequence: NC_000017.10.

**3. TA cloning using the pGEM-T Easy kit**

After transformation by JM109 competent cells to achieve high efficiency, the plates were incubated overnight at 37 ºC. White colonies were selected for culture in ampicillin-containing LB broth overnight. Each colony was purified using the AccuPrep Plasmid Purification Kit (Bioneer) and purified fragments were sequenced directly using forward or reverse primers (Supplementary Table 1) with the BigDye Terminator v3.1 Ready Reaction Kit (Applied Biosystems, Foster City, CA, U.S.A.).
